# Decoding the complexities of emotion socialization: cultures, individual features and shared information

**DOI:** 10.1038/s41598-024-52885-9

**Published:** 2024-02-12

**Authors:** Akira Takada, Elise Dan-Glauser

**Affiliations:** 1 Division of African Area Studies, Graduate School of Asian and African Area Studies (ASAFAS), Kyoto University, 46 Yoshida-Shimoadachi-cho, Sakyo Ward, Kyoto, 606-8304 Japan; 2 Institute of Psychology, Faculty of Social and Political Sciences, Unil-Mouline, University of Lausanne, 1015 Lausanne, Switzerland

**Keywords:** Human behaviour, Social behaviour

## Abstract

The ability to understand and regulate one’s emotions is an integral part of human development. As part of this learning process, emotion socialization is understood as the dynamic mechanism by which caregivers mediate and influence the child’s emotional competence. Failures in emotion socialization have been associated with antisocial behavior, peer rejection, and mental health issues in both children and adults, which underscores the importance of this process. It has been suggested that emotion socialization is strongly influenced by the socio-cultural features of the caregivers. This Collection compiles recent works that unravel the underlying complex mechanisms of emotion socialization and related life outcomes. It emphasizes the crucial role that cultural and individual traits play in the process of emotion socialization. Looking ahead, combining insights from neuro-physiological and socio-cultural perspectives promises to enrich our comprehension of emotional processes and emotional competence development.

Mastering emotions is a challenge for most human beings, and research has long focused on the underlying processes that drive emotions. Recent developments in neuroscience have provided a number of new insights into the neural and physiological basis of emotions. For example, a recent meta-analysis of neural-derived models of emotion regulation suggested a dynamic modulation of the connectivity between systems involved in the production of emotion and the systems involved in the regulation of emotion, specifically during cognitive emotion control, highlighting the robustness of task-modulated prefrontal-amygdala coupling^1^. On the other hand, arguments have been advanced on the socially and culturally constructed aspects of emotion. For example, Lisa Barrett's theory of constructed emotion describes emotion as the product of external states and internal states, which she calls core affect. In this categorization, the interpretation of internal states is fine-tuned by learned representations of emotions that rely on culturally embodied knowledge (see e.g.,^2^). The debate about the relative contribution of neuro-physiologically and socio-culturally constructed aspects of emotion has constituted opposing axes since the end of the nineteenth century, when the modern study of emotion began. It appears fundamental, however, to go beyond a conceptual battle and strive to integrate new findings from both theoretical stances to comprehensively deepen our understanding of emotion processes. In this context, the study of emotion socialization focuses on the process by which affects, which are physiological responses to external stimuli or changes in internal conditions, are transformed into socio-cultural emotions in the course of social life. In this respect, Barrett's theory of constructed emotion can be further developed from the perspective of both ontogenetic and socio-cultural development^3^. The articles in this Collection all provide recent results that feed the understanding of the importance and determinants of how the environment can shape the interpretation of our own emotions, informing on how biologically-based processes may be shaped at different levels by culture, caregivers and child features, and the type of content that is shared between individuals.

Starting at the highest meta-level, some studies examined how emotional displays may vary across cultures, portraying how mothers from different backgrounds convey their emotions differently to their children, and what kind of expression the child learns to display. With this aim, Abu Salih et al.^4^ investigated emotion communication and regulation strategies in mother and baby dyads and found that these were culturally distinct in Palestinian-Arab and Jewish dyads. Rochanavibhata and Marian^5^ further examined the collective-individualistic dimension by comparing how emotional displays between preschoolers and their mothers differ between Thai and US cultures, which are often characterized as collective and individualistic cultures, respectively. The results showed that American dyads expressed more intense emotions as opposed to Thai dyads. The findings of the study suggested that cultural values and norms may play a crucial role in shaping the way individuals express and regulate their emotions. The same study also tackled an important variable in emotion socialization, which is that of caregiver and child features. Hence, in Rochanavibhata and Marian's study described above, boy dyads showed, in both cultures, more emotionally intense expressions than girl dyads. Boys also displayed more negative emotional behaviors than girls during prompted reminiscing, whereas girls used more negative emotion words than boys during the personal narrative task.

Also examining gender, a study by Gergely et al.^6^ compared the characteristics of infant-, dog-, and adult-directed communication among Hungarian adults. The results indicate that both men and women tend to make happy expressions for their infants and dogs. The authors suggested that these features are formed to call and maintain the partner's attention, foster emotionally positive interactions, and strengthen social bonds with their partners. It will be interesting to clarify when and how different interactions, such as when communicating negative emotions, become possible, and how the behavior of infants, dogs, and adult partners in interactions affects the interaction itself. This leads us to reflect on the content of the interaction, which also plays a role in emotion socialization processes.

Regarding interaction content, Nikolic et al.^7^ examined how parental mental state, language, and warmth relate to shame and guilt in 2- to 5-year-old children. The results indicated that the combination of more parental mental state language, and high warmth was associated with a quicker willingness from the children to provide help to the experimenter. The authors argue that this is because language and warmth internalize and help children develop a sense of shame and guilt. As a draw to meta-variables (aka culture), the authors note that there is no guarantee that similar results would be obtained in societies where the sense of shame is supposed to be the foundation of cultural constructs (e.g., East Asia), as this study was conducted in a Western society (namely the Netherlands), where that of guilt is the foundation. Given the different purposes and uses of these different normative emotions in different cultures, comparative research on how the sense of shame and guilt is formed depending on the cultural context of the child is necessary. At the content level, we can also highlight the study by Arikan and Kumru^8^ in Turkey, which extends beyond the single content of the socialization process. This study goes a step further and examines the flexibility and diversification of communication at play. In this study, caregivers' emotion socialization strategies were characterized as unspecified (moderate in all emotion socialization strategies), supportive, and mixed (using both supportive and unsupportive strategies). From these studies, we can conclude that the issue of individual differences in caregivers' socialization strategies seems vital not only for how children are nurtured by them but also for how caregivers' mental health is maintained, which offers a dual perspective on the emotion socialization process, both from the infant and caregiver sides.

Taken together, the reported results in the papers published as part of this Collection suggest that the combination of cultural norms, gender norms, normative emotions, and interaction content and flexibility all significantly contribute to different aspects of emotion socialization. This Collection has further advanced our understanding of the cultural and individual differences in caregivers' emotion socialization strategies, their impact on caregivers' mental health, and the developmental characteristics and gender differences in children's emotion socialization strategies. Based on these contributions, we could however highlight current challenges in this field and comment on potential future directions. First, several articles in this Collection are cross-cultural comparisons of emotion socialization and deal with emotion socialization in non-British and non-American cultures as well. It would be beneficial to the field to continue gathering such a wide range of data while still improving the theoretical framework in order to better understand how cultural variations in emotion socialization arise. Second, research bridging emotion socialization and the self remains scarce, despite the fact that we think self-formation could be pivotal in emotion socialization. According to ^9^, self consists of two distinct units: the permanent self, which refers to a sense of consistent existence from the past to the future, and the temporary self, which refers to the sense that one's body belongs to oneself, that one's self is performing a certain action, and that it exists in one's immediate environment. Emotion socialization, influenced by repetitive social interactions, has long-lasting effects on individuals' emotional experiences and regulation strategies, which we believe shape their self-formation at both permanent and temporary levels. Hence, there may be a reciprocal relationship between emotion socialization and self-formation, and future research should consider both concepts when studying these processes. Third, the perspective of emotion socialization is also a powerful tool for advancing research on emotion-related disorders and disorder-related emotions. As an example of the first, findings indicate that depression symptomatology may be related to abnormalities in several systems of the organism, such as the autonomic, metabolic, or immune systems (see e.g.,^10,11^), which may be responsible for the depression-associated persistent fatigue, low motivation, and negative cognitions. Regarding disorder-related emotion functioning, people who exhibit Autism Spectrum Disorder (ASD) were shown to have reduced interoceptive accuracy but exaggerated interoceptive sensibility (see e.g.,^12,13^). People with ASD may thus have a stronger sense of emotion from their internal state than what is evaluated by objective measures. By purposely tackling such misalignment and focusing on internal state interpretation, research on emotion socialization can offer avenues for related interventions.

By exploring how physiological responses are transformed into socio-cultural emotions, emotion socialization is crucial to refining the biologically described emotion processes. The articles in this Collection examine how several features of our environment significantly shape the way we experience our internal state changes, and the way we learn to modulate them. In particular, it highlights the role of culture, caregivers, and the interaction content in this complex socialization process. The Collection highlights the importance of integrating findings from neuro-physiological and socio-cultural perspectives to deepen our understanding of emotions and socialization processes, notably by highlighting their pivotal role in human functioning.
